# Anatomy of Indian heatwaves

**DOI:** 10.1038/srep24395

**Published:** 2016-04-15

**Authors:** J. V. Ratnam, Swadhin K. Behera, Satyaban B. Ratna, M. Rajeevan, Toshio Yamagata

**Affiliations:** 1 Application Laboratory, Japan Agency for Marine-Earth Science and Technology, Yokohama Japan; 2 Ministry of Earth Sciences, Prithvi Bhavan, New Delhi, India

## Abstract

India suffers from major heatwaves during March-June. The rising trend of number of intense heatwaves in recent decades has been vaguely attributed to global warming. Since the heat waves have a serious effect on human mortality, root causes of these heatwaves need to be clarified. Based on the observed patterns and statistical analyses of the maximum temperature variability, we identified two types of heatwaves. The first-type of heatwave over the north-central India is found to be associated with blocking over the North Atlantic. The blocking over North Atlantic results in a cyclonic anomaly west of North Africa at upper levels. The stretching of vorticity generates a Rossby wave source of anomalous Rossby waves near the entrance of the African Jet. The resulting quasi-stationary Rossby wave-train along the Jet has a positive phase over Indian subcontinent causing anomalous sinking motion and thereby heatwave conditions over India. On the other hand, the second-type of heatwave over the coastal eastern India is found to be due to the anomalous Matsuno-Gill response to the anomalous cooling in the Pacific. The Matsuno-Gill response is such that it generates northwesterly anomalies over the landmass reducing the land-sea breeze, resulting in heatwaves.

Heatwaves affect the human comfort and are associated with marked short-term increases in mortality^1^. Studies^2^ have shown the urban areas across the globe to have significant increase in the number of heat waves during the recent decades. The recent increasing trend in the frequency and intensity of heatwaves is often attributed to climate change^2^,^3^,^4^. However, we need more detailed analyses because year-to year variations are large. The European heat wave of August 2003 caused total deaths of about 35000 with more than 14,800 deaths in France alone^1^. According to the report of Japan Ministry of Environment, deaths due to heat stroke during the unusually hot summer in 2010 amount to 1745 in Japan. Deaths were also reported in India due to heatwaves over the years. The heatwave of 1988 caused an estimated number of 1300 deaths^5^,^6^ and likewise the heatwaves of 1998 and 2003 caused deaths of about 2042 people^7^ and 3054 people^8^, respectively. According to EM-DAT^9^, the international disaster database, the heatwave of 2015 caused deaths of 2248 people in various parts of India. The heat waves over India are projected to be more intense and occur more frequently in future^4^. Climatologically heatwaves occur during March to June^10^,^11^, with high frequency over north, northwest, central and the eastern coastal regions of India.

The heatwaves over India have been linked with the climate modes such as El Niño-Southern oscillation (ENSO)^5^ and also to the variations in the sea surface temperatures in the Bay of Bengal^7^. Some studies also linked the heatwaves to the re-curving tropical cyclones in the Bay of Bengal^7^. The re-curving tropical cyclones before the onset of the heatwaves could change the direction of the winds and cut-off moisture to the inland regions leading to heatwaves. In spite of the large societal impact, there has been no systematic attempt to understand the principal mechanism of heatwaves over India. In this study we attempt to understand those causes based on observations and simple statistical analyses.

## Results

### Maximum Temperature Anomalies

**Figure 1:**
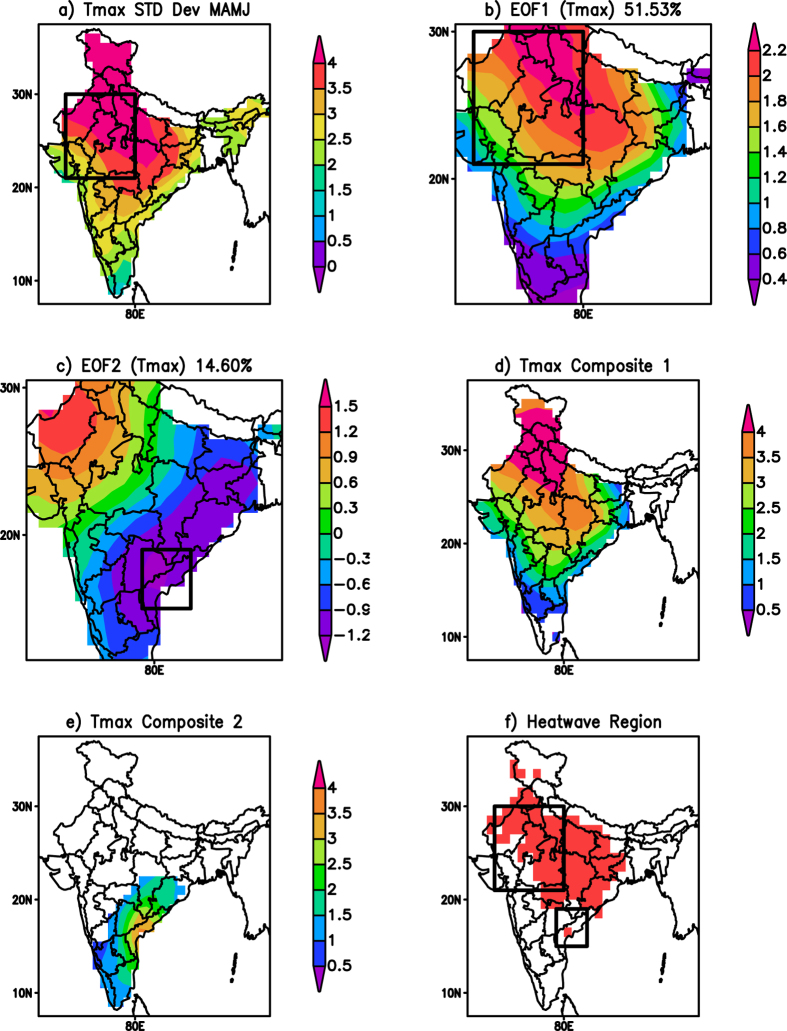
(**a**) Standard deviation of the maximum temperature (Tmax). (**b**) First mode of EOF of Tmax anomalies (**c**) The second mode of EOF of Tmax anomalies. (**d**) The composite of the Tmax (°C) anomalies corresponding to the heat wave events over north and central India. (**e**) same as (**d**) but for events over the coastal eastern India. (**f**) Spatial distribution of heatwaves over India. Only significant (at 95% using Students 2 tailed t-test) values are shown in (**d**,**e**). The rectangular box in (**a**–**c**,**f**) represents the region used to identify the heat wave events over India. (Figure was created using a free software Grid Analysis and Display System (GrADS) version 2.1.a3 (http://cola.gmu.edu/grads/downloads.php).

Standard deviation of daily maximum temperature (hereafter Tmax), from March to June over the years 1982–2013 (Fig. 1a), shows large values extending from north to central India apart from the coastal regions facing the Indian Ocean. Interestingly, the temperature anomalies during some of the recent heatwaves also show large positive departures in those regions. Moreover, the variability captured by the first mode of empirical orthogonal function (EOF) (Fig. 1b), which explains about 51.53% of the total variance, resembles the region of large standard deviation. These constitute the first-type of heatwave events here and the heatwave events are derived using the area averaged Tmax anomalies over the region 71^o^E–80^o^E; 21^o^N–30^o^N (box in Fig. 1a) corresponding to the region of large standard deviation. The Tmax anomalies are representative of the heatwaves which occur over north and central India. The Tmax anomalies derived from the area average index show a large correlation of 0.934 with the principal components of the first mode of EOF.

The heatwave events over the coastal regions of eastern India are derived from the Tmax anomalies averaged over the region 79^o^E–83^o^E; 15^o^N–19^o^N (box in Fig. 1c). This selected area is close to the southeast pole of the second EOF pattern (Fig. 1c), which explains about 14.6% of the total variance with a dipole structure in the Tmax anomalies. The correlation coefficient between the area averaged Tmax index for this second-type of heatwaves and the principal components of the second mode of EOF is −0.632. We identify significant heatwave events based on the criteria given in the methods section. Nineteen events are identified over the period 1982 to 2013 over the north and central India (Table 1). Similarly, thirteen events are identified over the coastal regions of eastern India (Table 1). The composite of the heatwave events for north and central India shows significant Tmax anomalies greater than 3 ^o^C over those regions (Fig. 1d). Those for the heatwaves in coastal eastern India are much weaker and significant Tmax anomalies confined to the coastal regions (Fig. 1e). To corroborate the choice of the regions for identifying the heatwave events, we computed the spatial distribution of heat waves over India (Fig. 1f) using the criteria given in the methods section. The spatial distribution (Fig. 1f) shows heatwaves to occur over northwest and central parts of India. Also a region of heatwaves is seen over the southeast coast of India (box near east coast of India in Fig. 1f). Comparing Fig. 1a,f, it can be seen that, most of regions affected by the heatwaves (Fig. 1f) are also the regions with large standard deviation (Fig. 1a) in the maximum temperature. Therefore, the two boxes identified for studying the heatwaves appropriately cover most of the the regions affected by heatwaves (Fig. 1a–c,f). We have also cross-checked by changing the domains of those boxes and found that the conclusions drawn here are not much affected by the choice of the domains for identifying the heatwave events.

### Heat Waves over north and central India

**Figure 2:**
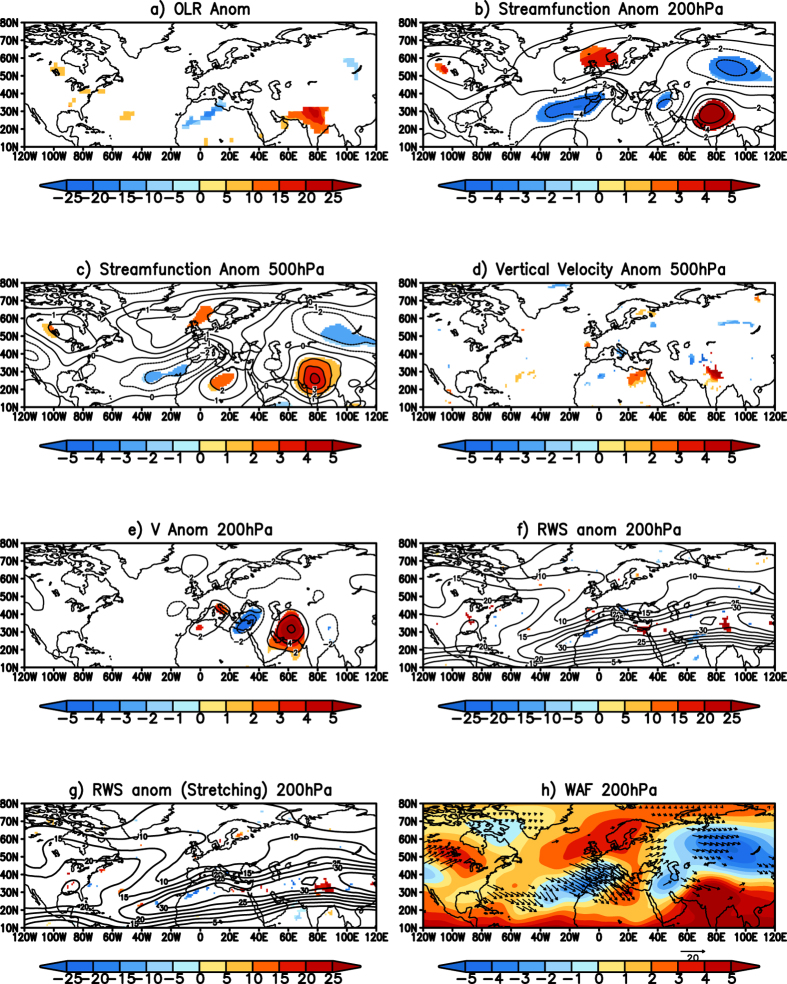
(**a**) Significant OLR (W/m^2^) anomalies of the composite of events over the north and central India. (**b**) same as (**a**) but for streamfunction (×10^6^ m^2^s^−1^) anomalies at 200 hPa. (**c**) same as (**b**) but at 500 hPa level. (**d**,**e**) same as (**a**) but represent the vertical velocity anomalies (Pa s^−1^) at 500 hPa and the meridional wind (m/s) anomalies at 200 hPa respectively. (**f**) same (**a**) but represent Rossby wave source anomalies (shaded) and zonal wind at 200 hPa (**g**) same as (**f**) but represents the Rossby wave source (×10^−11^ s^−2^) anomalies (shaded) due to stretching term and zonal wind. (**h**) Significant wave activity flux anomalies at 200 hPa (vector) and the streamfunction anomalies are shaded for the heatwave events over the north and central India. (Figure was created using a free software Grid Analysis and Display System (GrADS) version 2.1.a3 (http://cola.gmu.edu/grads/downloads.php).

The composite of outgoing longwave radiation^12^ (OLR) anomalies, a proxy to precipitation, during the heatwave events corresponding to the first-type, shows significant positive values over India (Fig. 2a) indicating the region to be cloud free and with no precipitation. The region of positive OLR anomalies corresponds to the region of large Tmax anomalies (Fig. 1d). The heatwaves over Europe are usually associated with strong atmospheric blockings in summer^13^. To see if a similar blocking phenomenon was the cause of the heatwaves over India, we plotted the eddy streamfunction anomalies at 200 hPa. The term “eddy” hereafter refers to departure from zonal symmetry. Composite of the 200 hPa eddy streamfunction anomalies shows a significant anticyclone over India (Fig. 2b). It is interesting to note that the anomalous anticyclone is a part of a quasi-stationary wave extending from the northwestern Africa (Fig. 2b). The significant anomalous cyclone over that region is located to the south of the anomalous anticyclonic blocking pattern seen over the North Atlantic Ocean (Fig. 2b). The wave pattern is seen at the level of 500 hPa (Fig. 2c) with a significant anticyclone over the Indian landmass, as well. The anomalous blocking pattern over the North Atlantic Ocean is seen clearly at the 500 hPa level (Fig. 2c). The anomalous cyclonic streamfunction to the west of North Africa is associated with the anomalous blocking pattern seen over the North Atlantic Ocean (Fig. 2c). The vertical velocity at 500 hPa shows anomalous sinking (Fig. 2d) over the Indian landmass due to the anomalous anticyclone over the region (Fig. 2c); the sinking motion causes positive OLR anomalies (Fig. 2a), resulting in heatwaves over the region. On the other hand, anomalous rising motion is seen over northwestern North Africa (Fig. 2d) due to the cyclonic anomalies (Fig. 2c) seen over the region leading to the negative OLR anomalies (Fig. 2a). The quasi-stationary wave pattern from North Africa to India is clearly seen in the meridional wind anomalies at 200 hPa (Fig. 2e) corroborating the wave-train identified seen in the streamfunction anomalies (Fig. 2b).

To look at the cause of the anomalous quasi-stationary Rossby wave pattern, we have calculated the Rossby wave source^14^,^15^ (RWS) anomalies at the level of 200 hPa for all the events using equation (1)^15^


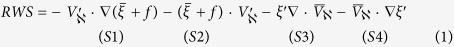


where, the term S1 represents the advection of the vorticity by the divergent anomaly winds and the S2 term represents the stretching of the vorticity by the divergent anomalies. The terms S1 and S2 contribute most to the RWS anomalies^15^. The terms S3 and S4 represent the advection and stretching due to the anomalies.

The RWS anomalies in tropical processes are mainly generated due to the advection of the mean absolute vorticity by the anomalous divergent winds (term S1 of equation (1)) and the extratropical RWS anomalies are due to the stretching of the absolute vorticity by the anomalous divergent winds (term S2 of equation (1))^14^,^15^. The composite of the RWS anomalies during the heatwave events over India shows a significant source over North Africa (Fig. 2f; shaded). Interestingly, the RWS anomaly is located at the entrance of the African Westerly Jet (Fig. 2f; contour). The anomalous Rossby waves generated due to the anomalous RWS at the entrance of the Jet can easily propagate along the Westerly Jet, which acts as a waveguide for the propagation^16^,^17^,^18^. The separation of the RWS anomalies into its components shows that the anomalous stretching of the absolute vorticity by the anomalous divergent winds (term S2 of equation (1)) (Fig. 2g; shaded) has the largest contribution to the RWS anomalies (Fig. 2f) during the heatwave events. The wave activity flux^19^, a diagnostic exhibiting the propagation of Rossby waves, shows an anomalous quasi-stationary Rossby wavetrain generated due to the RWS anomalies over North Africa. The wavetrain then propagates along the Westerly Jet (Fig. 2h). The phase of the quasi-geostrophic Rossby wave is such as to cause an anomalous anticyclone over India. The anomalous anticyclone, which is seen extending to 500 hPa (Fig. 2c), suppresses convection leading to heatwave conditions over that region. The analysis shows that the heatwaves over north and central India are due to the anomalous quasi-stationary wave originating at the entrance of the African Jet. The anomalous wave pattern seems to be generated by the anomalous blocking in the North Atlantic Ocean.

We further validated the above hypothesis, that the blocking over North Atlantic Ocean was the cause of the heatwaves over north and central parts of India, by computing the Granger causality^20^ test. To check if the blocking events in North Atlantic Ocean can Granger cause heat waves over India we carried out Granger causality tests between the daily 200 hPa geopotential height anomalies (Z200 hereafter) averaged over the region 50^o^N–75^o^N; 40^o^W–20^o^E, the region with significant anticyclonic anomaly in North Atlantic (Fig. 2b), and the daily Tmax anomalies averaged over the region 71^o^E–80^o^E; 21^o^N–30^o^N, the region used to identify heatwave events. Both time series are normalized by their standard deviation. Applying the KPSS^21^ stationarity test to both the normalized time series showed the normalized Tmax time series to be nonstationary. The time series are made stationary, which is essential for applying the Granger causality test, by applying a first difference operator to both the time series^22^,^23^. Granger causality test is first applied to the time series to see if the prediction of Tmax anomalies (say y) improves by including the 200 hPa geopotential anomalies (say x) as predictor at various time lags. The second test is to see if the Tmax anomalies as predictor can be used to improve the forecasts of 200 hPa geopotential anomalies. In the first test Tmax is the response variable and 200 hPa geopotential anomalies is the response variable in the second test. If the first test is statistically significant and the second one is not, then we can say that 200 hPa geopotential anomalies Granger cause Tmax anomalies at that particular lag. The tests are a Wald test comparing a full model, in which y is explained by the lags of both y and x, and the restricted model, in which y is only explained by the lags of only y. Statistical significance tests are to test the null hypothesis that the restricted model is adequate against the full model. Table 2, shows the results of Granger causality test at lags from 1 to 5. Table 2 shows that the null hypothesis is rejected for a lag of 2 at significance level of less than 0.05 using F-test, with Tmax anomalies as the response function, indicating that the lagged values of 200 hPa geopotential anomalies improve the prediction of Tmax anomalies. In contrast, for lag 2, the null hypothesis fails for the test with 200 hPa geopotential anomalies as the response function. These results show that the 200 hPa geopotential anomalies over North Atlantic Granger cause Tmax anomalies over India with a lag of 2 days. For the other lags of 1, 3, 4, 5 days (Table 2) the results fail to reject the null hypothesis with both Tmax and 200 hPa geopotential anomalies as the response functions. The above results show that the blocking events in North Atlantic Ocean can cause heatwave events over north and central parts of India.

### Heat waves over coastal eastern India

**Figure 3:**
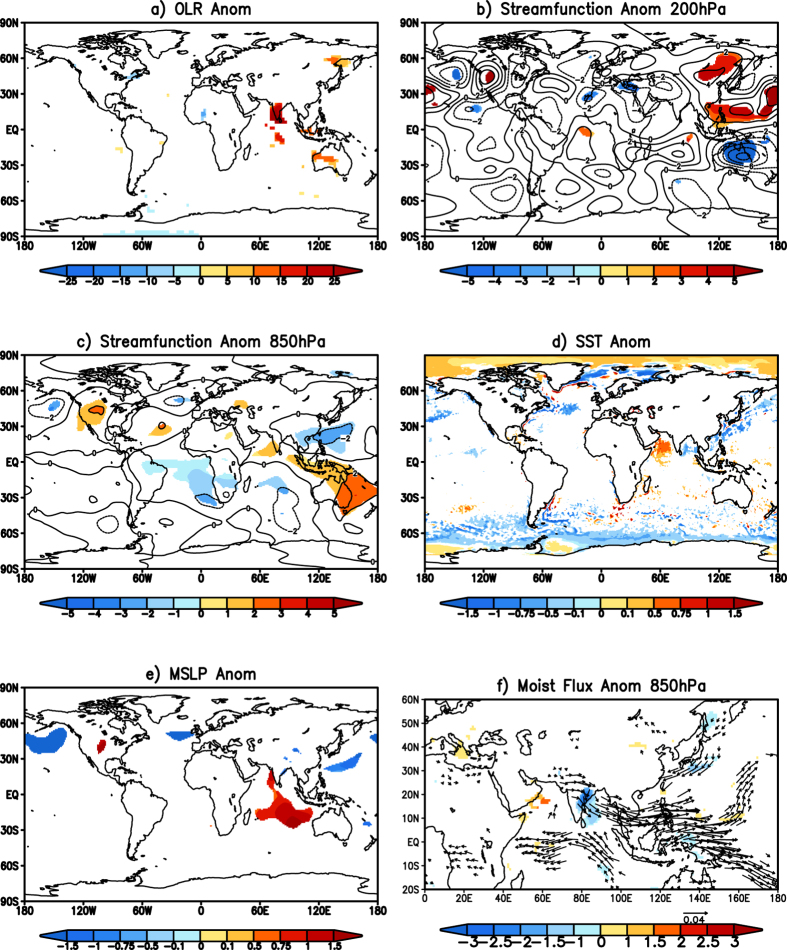
(**a**) Significant OLR (W/m^2^) anomalies of the composite of events over the east coast of India. (**b**,**c**) same as (**a**) but for streamfunction (×10^6^ m^2^s^−1^) anomalies at 200 hPa and 850 hPa respectively. (**d**,**e**) same as (**a**) but for significant SST (°C) and Mean sea level pressure (mb) anomalies. (**f**) same as (**a**) but for the significant moisture flux (kg m^−1^ s^−1^) anomalies (vector) and the specific humidity (kg/kg) anomalies (shaded) at 850 hPa. (Figure was created using a free software Grid Analysis and Display System (GrADS) version 2.1.a3 (http://cola.gmu.edu/grads/downloads.php).

The composite of OLR anomalies for the events corresponding to the second type of heatwaves shows significant positive values over south India and along the coastal eastern India (Fig. 3a) corresponding to large significant Tmax anomalies (Fig. 1e). This indicates that the region is cloud free. To understand processes which are responsible for the heatwaves of the second type, the composite eddy streamfunction anomalies at 200 hPa (Fig. 3b) are plotted. The 200 hPa composite eddy streamfunction anomalies shows a pair of anticyclonic anomalies over the west Pacific similar to the Matsuno-Gill^24^,^25^ response to the tropical Pacific cooling. The eddy streamfunction anomalies do not have significant values over the Indian region, indicating that the heatwaves over the east coast of India are not caused by the quasi-stationary Rossby waves like the heatwaves over the central and north India. The composite of eddy streamfunction at 850 hPa (Fig. 3c) shows the baroclinic Matsuno-Gill response extend to low levels with cyclonic cells straddling the equator over the west Pacific. A significant anticyclone is also seen over the southern tip of India, which indicates the westerly winds driven out of the Indian landmass. The composite of the mean sea surface temperature anomalies (Fig. 3d) shows significant negative anomalies over the equatorial central-east Pacific and positive anomalies over the west Pacific, though the regions of the significant anomalies are small in extent. The mean sea level pressure anomalies of the composite (Fig. 3e) show an anomalous low-pressure area along the east coast of India indicating the winds blowing out of India. The 850 hPa moisture flux anomalies (Fig. 3f) shows the westerly transport of moisture out of India and transported to the cyclonic anomalies (Fig. 3c) in the west Pacific. The transport of the moisture out of the Indian landmass significantly reduces the specific humidity (Fig. 3f) and causes the reduction in precipitation along the east coast of India.

**Figure 4:**
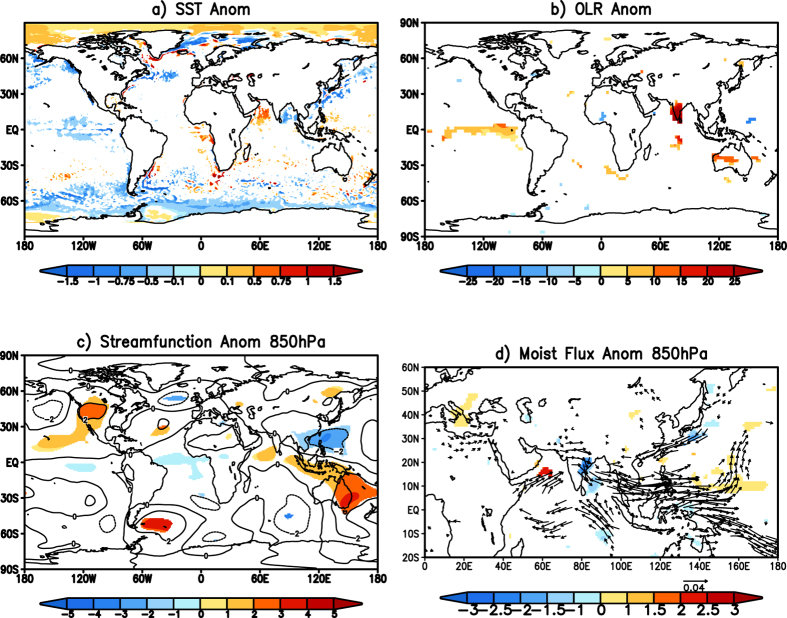
(**a**) Significant SST (°C) anomalies for the events over the east coast of India. (**b**) same as (**a**) but for OLR (W/m^2^) anomalies. (**c**) same as (**a**) but for streamfunction (×10^6^ m^2^s^−1^) anomalies at 850 hPa. (**d**) Moisture flux (kg m^−1^ s^−1^) anomalies (vector) and specific humidity (kg/kg) anomalies (shaded) at 850 hPa. (Figure was created using a free software Grid Analysis and Display System (GrADS) version 2.1.a3 (http://cola.gmu.edu/grads/downloads.php).

On analyzing the SST anomalies of the individual events, it is found that the event of 1983 and 1997 (Table 1) were associated with positive SST anomalies in the equatorial Pacific. To get a clear picture of the process that would have caused the heatwaves over east coast of India, we have made a composite of the remaining 11 events (Fig. 4). The composite of the events shows significant negative SST anomalies over the east Pacific though over a wider region (Fig. 4a). The OLR anomalies associated with the negative SST anomalies show significant positive values from the central Pacific to east Pacific (Fig. 4b). The Matsuno-Gill response to the cooling over the equatorial pacific results in a pair of significant anomalous cyclonic cells straddling the equator (Fig. 4c) over the west Pacific. The composite of the anomalous moisture flux again shows moisture being transported out of India towards the cyclonic cells in the west Pacific (Fig. 4d). The anomalous transport of moisture reduces the humidity over the Indian landmass, results in anomalous reduction of specific humidity over the east coast of India (Fig. 4d) and the associated heatwaves over the region. The land-sea breeze, which is the major source of moisture for the coastal regions, is reduced due to the anomalous circulation pattern related to the tropical Pacific conditions. The reduction of moisture and convective activity over the region results in increase of temperatures hence the heatwaves.

We used Granger causality tests between the daily 850 hPa eddy streamfunction anomalies (SF850 hereafter) averaged over the region 10^o^N–30^o^N; 100^o^E–150^o^E, the region with significant cyclonic anomaly in west Pacific (Fig. 4b) and the daily Tmax anomalies averaged over the region 79^o^E–83^o^E; 15^o^N–19^o^N to further verify our results. Both time series are normalized by their standard deviation. Applying the KPSS^16^ stationarity test to the normalized time series shows both the time series to be stationary. Table 3, shows the results of Granger causality test between Tmax and SF850 at lags from 1 to 5. Table 3 shows that the null hypothesis is rejected for lag equal to 1 at significance level of less than 0.05 with Tmax as the response function, indicating that the lagged values of SF850 improve the prediction of Tmax. In contrast, for lag value of 1, the null hypothesis fails for the test with SF850 as the response function. These results show that the 850 hPa streamfunction anomalies over west Pacific Granger cause Tmax anomalies over India with a lag of 1 day. For the other lags of 2, 3, 4, 5 days (Table 2) the results reject the null hypothesis with both Tmax and SF850 as the response function indicating that the two variables do not have causal relation at those lags.

### Heatwave event of 2015

We take up a case study of heatwave which occurred during May 2015 to see if the processes discussed in the previous sections were important for the maintenance of the heatwave. During the third and fourth week of May 2015 (21^st^ May 2015 to 31^st^ May 2015), India experienced heatwave^26^,^27^,^28^, killing at least 2248 people in various parts of India. Averaged daily maximum temperatures were high over northwest India, central India and along the southeast coast of India^27^,^28^ during the period of the heat wave. The distribution of the maximum temperature shows that the heat wave of 2015 was of the first type discussed in previous section, with heat wave conditions over north and central India.

**Figure 5:**
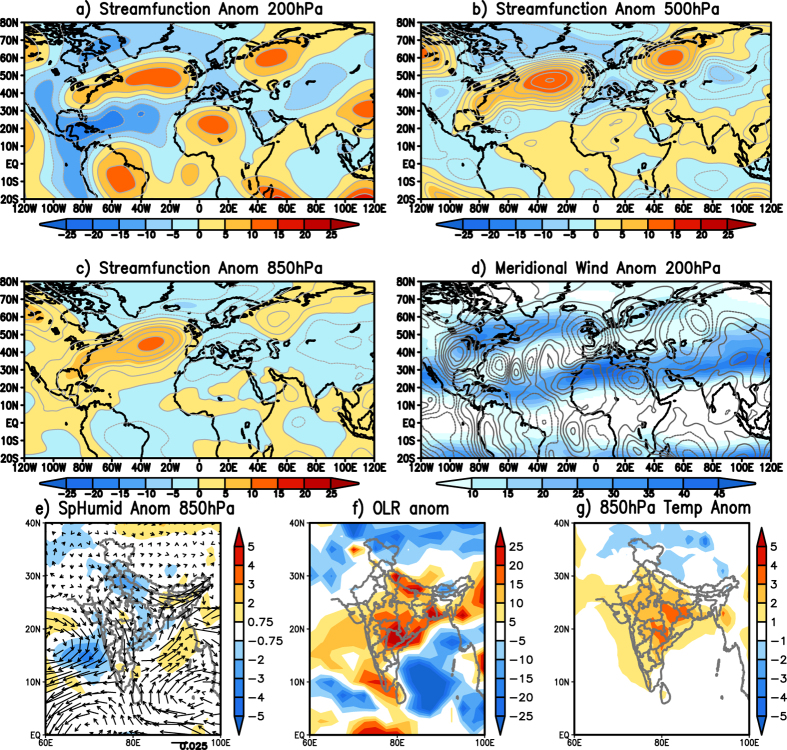
(**a**) Eddy streamfunction (×10^6^ m^2^s^−1^) anomalies at 200hPa averaged over 21^st^ May2015 to 31 May 2015. (**b**,**c**) same as (**a**) but at 500 hPa and 850 hPa respectively. (**d**) 200 hPa meridional wind anomalies (contour) and zonal wind (shaded) averaged over 21^st^ May 2015 to 31^st^ May 2015. (**e**) same as (**d**) but 850 hPa specific humidity (Kg/Kg) anomalies (shaded) and moisture flux (kg m^−1^s^−1^) anomalies (vector). (**f**,**g**) same as (**d**) but OLR and 850 hPa temperature anomalies respectively. (Figure was created using a free software Grid Analysis and Display System (GrADS) version 2.1.a3 (http://cola.gmu.edu/grads/downloads.php).

Eddy stream function anomaly (200 hPa) averaged over 21^st^ May 2015 to 31^st^ May 2015, shows anticyclonic anomaly over India (Fig. 5a). Anomalous atmospheric blocking and the associated cyclonic anomaly to the south of it can be clearly seen in North Atlantic Ocean during the heatwave period (Fig. 5a). A wave train from west Africa can be clearly seen in the meridional wind anomalies at 200 hPa (Fig. 5d; contour) along the westerly jet (Fig. 5d; shaded), contributing to the anticyclonic anomaly over India. The anomalous anticyclonic blocking over North Atlantic Ocean can also be seen at 500 hPa (Fig. 5b) and 850 hPa (Fig. 5c) levels. The anticyclonic anomaly over India extends up to 500 hPa (Fig. 5b) and is seen as cyclonic anomaly at 850 hPa (Fig. 5c). The cyclonic anomaly over India is seen covering parts of northwest India, central India and parts of east coast of India (Fig. 5c). The anomalous cyclone over India (Fig. 5c) is seen transporting anomalously dry air (Fig. 5e) from northwest India into interior parts of India. The transport of anomalously dry air from the northwest anomalously reduces moisture over northern parts of India and along the southeast coast of India (Fig. 5e). The reduction of moisture is also evident from the positive OLR anomalies (Fig. 5f) observed during the period of heatwave in May 2015. Positive OLR anomalies over India, which are proxies to precipitation, indicate anomalously cloud free region. The solar radiation anomalously increases in the cloud free regions increasing temperatures (Fig. 5g) over India. Our analysis showed that the anomalous blocking over North Atlantic to be the cause of the heat waves over India during May 2015. The processes causing the heatwave in 2015 are similar to the processes discussed in the composite of the heatwaves over north and central India.

## Discussion

In the present study we tried to understand the processes maintaining the heatwaves over India. Based on the standard deviation of the Tmax and on the EOF analysis we classified the heatwaves over India into two types, those that occur over the north-central India and those that occur over the coastal eastern India. Our analysis showed that the first type of the heatwave, over the north-central India, to be associated with anomalous blocking over the North Atlantic Ocean. The anomalous blocking over North Atlantic Ocean results in a cyclonic anomaly west of North Africa at upper levels. Analysis of the Rossby wave source anomalies shows that the stretching of vorticity, west of North Africa, generates a Rossby wave source of anomalous Rossby waves near the entrance of the African Jet at 200 hPa. The resulting quasi-stationary Rossby wave-train along the Jet has a positive phase over Indian subcontinent causing anomalous sinking motion and thereby heatwave conditions over India. A case study of heatwave of May 2015, which caused loss of lives in India showed that the anomalous blocking over North Atlantic Ocean played an important role in the maintenance of the heat wave over India.

The second-type of heatwave over the coastal eastern India is found to be associated with the the anomalous baroclinic Matsuno-Gill response to the anomalous cooling in the Pacific. The Matsuno-Gill response at 850 hPa shows a pair of cyclonic anomalies across the equator in the west Pacific. The cyclonic anomaly in the north-west Pacific helps generate westerly anomalies over the landmass, which reduces the land-sea breeze along the coastal regions, resulting in heatwaves.

With the frequency of heatwaves to increase in near future^2^,^3^,^4^, the findings of this study will be beneficial to the society.

## Methods:

### Identification of heat wave events

The high resolution gridded temperature daily maximum temperature data set of the India Meteorological Department (IMD)^29^ is used for identifying the heat wave events in the study. The data is available from 1951 to 2013. We used the data from the satellite era, 1982 to 2013 for identifying the heat wave events over India. The daily Tmax anomalies are derived based on the daily climatology from 1982 to 2010. The heatwave events are identified using the Tmax anomalies satisfying the criteria set by the IMD. For identifying the heatwaves over north and central India, the Tmax anomalies from 1^st^ March to 30^th^ June over the period 1982–2013 are area averaged over the region 71^o^E–80^o^E; 21^o^N–30^o^N. The area averaged time series is normalized by its own standard deviation. We pick up the heatwave events when the normalized area average Tmax anomalies are greater than one standard deviation for 6 days or more consecutively. The spatial distribution of the Tmax anomalies is checked for all the days of the event to satisfy the criteria suggested by the IMD. The criteria used by IMD^8^,^30^ in identifying the heatwaves is:

(i) Heatwave need not be considered till maximum temperature of the stations reached at least 40 ^o^C for plains and at least 30 ^o^C for hilly regions.

 (ii) When normal maximum temperature of a station is less than or equal to 40 ^o^C: A departure of 5 ^o^C to 6 ^o^C from normal is to be considered a heatwave.

(iii) When the normal maximum temperature of a station is more than 40 ^o^C: A departure of 4 ^o^C to 5 ^o^C from normal is to be considered a heatwave.

 (iv) When actual maximum temperature remains 45 ^o^C or more irrespective of normal maximum temperature heat wave should be declared.

  (v) For coastal stations if the maximum temperature of 40 ^o^C is reached, heat wave may be declared.

The heatwaves over the east coast of India are identified using the normalized Tmax anomalies area averaged over the region 79^o^E–83^o^E; 15^o^N–19^o^N. The events satisfy the criteria that the normalized area averaged Tmax anomalies be greater than one standard deviation for 6 days or more and the spatial distribution of the Tmax anomalies satisfy the IMD criteria for heat wave on all the days of the event. To avoid duplication, the events that are covered in the heat waves over north and central India are not considered in the heatwave events over the east coast of India.

For calculating the spatial distribution of heatwaves over India, daily Tmax anomalies over each grid point are normalized by their standard deviation. A heatwave event is set to have occurred at a grid point if the normalized Tmax anomalies are greater than one standard deviation for 6 or more days consequently and also the criteria set by IMD is also satisfied.

The EOF used in the study is derived by considering the daily Tmax anomalies from March to June from 1982 to 2013, over the region 12^o^N–30^o^N; 70^o^E–90^o^E.

ERA-Interim reanalysis^31^ dataset at a resolution of 1deg is used to study various aspects of the heat waves. Daily anomalies of various fields are derived from daily climatology based on 1982 to 2010. Significance of the composite of events is tested using students 2 tailed t-test at 95% level.

## Additional Information

**How to cite this article**: Ratnam, J. V. *et al.* Anatomy of Indian heatwaves. *Sci. Rep.* **6**, 24395; doi: 10.1038/srep24395 (2016).

## Figures and Tables

**Table 1:** List of heat wave events over north and central India and the heat wave events over the coastal eastern India.

Heat wave events over north and central India		
19–30 May 1984	29 may–4 Jun 1994	18–27 Jun 2009
24–30 Jun 1987	30 may–10 Jun 1995	18–27 Mar 2010
8 May–14 May 1988	21 May–28 May 1998	8–20 Apr 2010
26–31 May 1988	7–12 Apr 1999	12–26 May 2010
11–21 Jun 1992	30 Apr–5 may 1999	18–24 May 2013
2–8 May 1993	16–24 Mar 2004	
7–13 Jun 1993	15–22 Jun 2005	
Heat wave events over the east coast of India		
29 May-4 Jun 1983	10 May–17 May 1996	14 May–20 May 2008
01 May-07 May 1985	29 May–07 Jun 1997	26 May–03 Jun 2010
07 Jun-13 Jun 1986	09 May–15 May 2002	30 May–06 Jun 2012
17 May-23 May 1986	25 May–13 Jun 2003	
7 May-13 May 1994	15 May–21 May 2007	

**Table 2:** Results from a series of Granger causality tests using Tmax and Z200.

Lag (L)	F Tmax_response_	Pr(>F Tmax_response_)	F Z20_response_	Pr (>F Z200_response_)
1	2.5706	0.109	1.3425	0.2467
2	3.1319	0.04375*	2.2113	0.1097
3	2.5576	0.05344	1.6069	0.1856
4	2.1037	0.07773	1.6647	0.1553
5	1.7812	0.1131	1.6815	0.1355

Lag refers to the maximum number of lags used in the models. F Tmax _response_ (Pr(>F Tmax _response_) is the value of the F-statistic when Tmax(Z200) is the response variable. Pr(>F Tmax _response_) [Pr (>F Z200_response_)] is the probability of observing a value of F equal to or exceeding this value from an F distribution. Starred value is significant at less than 0.05 level.

**Table 3:** Same as Table 2 but results of Granger causality test between Tmax and SF850.

Lag(L)	F Tmax_response_	Pr(>F Tmax_response_)	F SF850_response_	Pr (>F SF850_response_)
1	26.727	2.461*1e–7*	0.2316	0.6303
2	13.912	9.541*1e–7*	3.2335	0.0394*
3	9.2735	4.18*1e–6*	3.1331	0.02454*
4	7.3852	6.37*1e–6*	2.5467	0.03756*
5	6.0388	1.417*1e–5*	1.9189	0.08784

Starred values are significant at less than 0.05 level.
